# WSNs Data Acquisition by Combining Hierarchical Routing Method and Compressive Sensing

**DOI:** 10.3390/s140916766

**Published:** 2014-09-09

**Authors:** Zhiqiang Zou, Cunchen Hu, Fei Zhang, Hao Zhao, Shu Shen

**Affiliations:** 1 Nanjing University of Posts and Telecommunications, Nanjing 210003, China; E-Mails: duihuhu@163.com (C.H.); zfcsat@126.com (F.Z.); wjzhh815@163.com (H.Z.); shens@njupt.edu.cn (S.S.); 2 Jiangsu High Technology Research Key Laboratory for Wireless Sensor Networks, Nanjing 210003, China; 3 Department of Geography, University of Wisconsin-Madison, Madison, WI 53706, USA

**Keywords:** compressive sensing, wireless sensor networks, sparse representation, hierarchical routing method, energy efficiency

## Abstract

We address the problem of data acquisition in large distributed wireless sensor networks (WSNs). We propose a method for data acquisition using the hierarchical routing method and compressive sensing for WSNs. Only a few samples are needed to recover the original signal with high probability since sparse representation technology is exploited to capture the similarities and differences of the original signal. To collect samples effectively in WSNs, a framework for the use of the hierarchical routing method and compressive sensing is proposed, using a randomized rotation of cluster-heads to evenly distribute the energy load among the sensors in the network. Furthermore, *L1*-minimization and Bayesian compressed sensing are used to approximate the recovery of the original signal from the smaller number of samples with a lower signal reconstruction error. We also give an extensive validation regarding coherence, compression rate, and lifetime, based on an analysis of the theory and experiments in the environment with real world signals. The results show that our solution is effective in a large distributed network, especially for energy constrained WSNs.

## 1. Introduction

Wireless sensor networks (WSNs) are used in a variety of applications, such as environmental data collection, dangerous event monitoring, and disaster prevention [1,2]. However, they have some performance limits (e.g., energy consumption). Conventional WSNs protocols, like location-based protocols [3] and hierarchical protocols [4], are helpful in reducing bandwidth requirements. The theory of compressive sensing (CS) [5,6], a novel sensing/sampling paradigm that goes against common wisdom in data acquisition, can further reduce the bandwidth requirements and save more energy.

Candès and Wakin [5] provided an introduction to compressive sampling, which is usually used in the field of efficient digital image compression. If a signal is known to be compressible, Donoho [6] found that the number of measurements *M* for this signal is dramatically smaller than the size of signal *N* (*M* ≪ *N*). Note that a signal being compressible means that it can be exploited to hold the correlation of the data both temporally and spatially based on some sparsification bases.

A number of papers focus on the combination of CS theory and WSNs [1,7,8]. Fazel *et al.* [1] proposed a distributed energy-efficient sensor network scheme based on random access compressed sensing. However, the authors in [1] do not address the scenario of multi-hop wireless networks. A recent survey of CS theory as applied in WSNs is given in [7], which recovers sparse data in WSNs by solving a convex optimization via *L1* norm. The authors in [8] discussed EW-CS scheme that is better overall recovery quality for non-uniform compressible signals than ordinary CS schemes. However, it is not feasible to simply combine CS theory with WSNs since the recovery procedure would fail due to the coherence between measurement and sparsity matrices in a real WSN scenario as described later and reference [9].

The research group of Xiang, Luo, Vasilakos and Rosenberg has done great work in the field of data collection in wireless sensor networks [9–12]. Reference [9] illustrated two crucial insights: firstly, applying CS naively may not bring any improvement, which coincides with our viewpoint in this paper, such as the bad performance of *L1* described in Table 1; and secondly, they put forward the idea that the hybrid-CS can achieve significant throughput improvements. Based on their previous hybrid-CS, reference [10] further proposed two solutions for data collection, *i.e.*, the optimal solution and the near-optimal solution. Reference [11] proposed the Dual-lEvel Compressed Aggregation framework to recover the physical signal from incomplete data from WSNs, which is also able to be done from our HRM_CS. The amazing work of their group is the CS-based aggregation scheme, which achieves both recovery fidelity and energy efficiency in WSNs with arbitrary topology according to the reference [12]. They employed diffusion wavelets to design the sparse basis while we focus on the recovery method based on Bayesian Compressed Sensing and the problem of coherence in the model of HRM_CS, which is more practical under the environment with real world signals.

Quer *et al.* [13] proposed a sparsity model that allows the use of CS theory for the online recovery of large data sets. While some of our work was inspired by the study reported in [13], it not only extends the results given in [13] by using the hierarchical routing method, but also provides a mathematical analysis of sparsity and coherence. In our previous paper [2], we proposed a data fusion method based on CS, which was used to monitor the cyanobacteria bloom-forming in a lake using a single hop network.

In the past years, considering the energy consumption in the procedure of route, reference [14] put forward a recurrent neural network to realize the range-free localization of WSNs while we decrease the energy consumption by making use of compressive sampling. These are two different methods to solve a similar problem. Joining CS theory with a routing free algorithm would be a worthwhile topic for future work.

The main existing problems that limit widespread applications under the real WSN scenario are as follows: (1) lack of a suitable method for a large region covered by a multi-hop network; (2) lack on an effective analysis of sparsity and coherence; and (3) lack of a quantitative analysis of the lifetime of the whole WSN. In this paper we solve all of these issues by jointly employing the hierarchical routing method and compressive sensing in multi-hop networks. The main contributions of this paper are the following:

- A model combining the hierarchical routing method and compressive sensing (called the HRM_CS) for WSN data acquisition, which includes some variant models, e.g., a model combining the hierarchical routing method and the Bayesian compressed sensing (BCS) based CS recovery algorithm (HRM_CS1), and a model combining the hierarchical routing method and the *L1*-minimization based CS recovery algorithm (HRM_CS2).
- A quantitative mathematical analysis of the HRM_CS, which includes signal sparsity analysis, measurement matrix analysis, coherence analysis, and so on.
- Design of a HRM_CS framework, being used for the online recovery of large data sets by collecting a small number of readings.
- Proof of the effectiveness of our approach for the acquisition and recovery of signals measured in an actual WSN deployment.

The paper is structured as follows: in Sections 2 and 3, we present our HRM_CS model for WSN data acquisition and the corresponding analysis in terms of signal sparsity, signal recovery, and routing method. In Section 4, we provide a framework for implementing the HRM_CS. In Section 5, through comparison with standard approaches, we prove the effectiveness of our model. Finally, we present our conclusions in Section 6.

## 2. WSNs Data Acquisition Model

In this section we firstly review basic CS theory from a sparse signal model and recovery model. Then we present the hierarchical routing method. Finally, we introduce our HRM_CS model for WSN data acquisition, through the joint use of the hierarchical routing method and compressive sensing.

### 2.1. Compressive Sensing Theory

Consider a grid WSN, consisting of *N* (*N* = *I* × *J*) sensors located on a two-dimensional plane for monitoring an environment with *I* and *J* sensors in the *x*- and *y*-directions, respectively. Each sensor in the network grid independently acquires a measurement and transmits this data towards the sink along the routing path. We firstly transform the two-dimensional data matrix ***U*** of the original signal into one-dimensional data vector ***X*** by Equation (1):

(1) $$\text{X}=\text{Vec}(\text{U})={\left[u_{11}\ldots u_{I1}\ldots u_{12}\ldots u_{I2}\ldots u_{1J}\ldots u_{IJ}\right]}^{T},\text{X}\in R^{N}$$

Taking into account the sparsity of natural phenomena (e.g., the temporal correlation and spatial correlation) in the monitored environment [1,2], based on classic compressive sensing theory, we can create *M* random projections of ***X***, that is, ***Y*** ∈ *R^M^*, and obtain the following mathematical model:

(2) $$\{\begin{array}{ll} \chi & =\Psi^{-1}\text{X},\text{X}=\Psi \chi ,{\left\|\chi \right\|}_{0}=K,K\ll N\\ \text{Y} & =\Phi \text{X}+\text{Z}=\Phi \Psi \chi +\text{Z},\\ \Phi & ={\left\{\phi_{1},\phi_{2},\ldots ,\phi_{M}\right\}}^{T},\phi_{i}\in R^{N} \end{array}$$

where ***Φ*** is the measurement matrix, which is referred to as the routing matrix since it indicates the way in which our sensor data is acquired and transmitted to the sink; ***Ψ*** is an invertible transformation *N* × *N* matrix; *N* > *M* ≥ *C_K_* · *K* · long *N* with a constant *C_K_*; original signal ***X*** is called *K*-sparse if it has only *K* significant components; ***χ*** is the sparse representation of ***X***; and ***Z*** ∈ *R^M^* is a vector denoting a noisy signal from *M* sensor nodes after the random measurement. Considering the computation capability of WSNs, we let sink node with no energy constraints and high computing capability to complete the complex matrix computation while let each sensing node only to sensing the physical signal.

#### 2.1.1. Signal Sparsity

Various common transforms can be used in the sparse signal model, such as Haar wavelets, the Fourier transform, principal component analysis (PCA) [15–17], and the discrete cosine transform (DCT). In the following, considering a real world signal [18], we focus on the latter two transforms, *i.e.*, PCA and DCT, which are used in the later model. Based on matrix algebra, in order to identify patterns in data and express the data in such a way as to highlight their similarities and differences in ***X***, we apply PCA to find patterns in the data and compress them, *i.e.*, by reducing the number of dimensions, without much loss of information. First, we calculate the eigenvectors and eigenvalues of the covariance matrix. Then, we choose components to form a feature vector. DCT is another transform [19,20], defined as ***Ψ*** ∈ *R^N^*^×^*^N^*, ***Ψ****^T^* ***Ψ*** = *E*. In our previous work, we used the observed chlorophyll-A data from the sensor nodes located in the Taihu Lake in China (longitude: 120.296, latitude: 31.387) as the original signal and applied DCT as a transform. From the results given in [2], almost 99% of the energy of size *N* = 92 is contained in only six important coefficients, *i.e.*, *K* = 6.

#### 2.1.2. Signal Recovery

Based on the previous work, if the original signal is sparse, it can be recovered with high probability using a method with some optimization techniques, such as *L1*-minimization [2,5,6] and BCS [21–23]. With a sufficient number of measurements, the sink is able to reconstruct the sensor readings by solving an *L1*-minimization problem [5,6]. By using *L1*-minimization technology [2], we reconstructed the chlorophyll-A signal from a smaller sample with higher accuracy; the mean of the reconstruction error for sampling point-1 was 0.17%, while that for sampling point-2 was 1.59%. As for the BCS method [23], its basic idea is to maximize the posterior from a Bayesian perspective, estimate sparse coefficients from the Bayesian framework, and then compute the estimated value of the original signal. In BCS, the estimated parameters are random variables with some prior distribution. By learning the *i*th sample *x^i^* and the Bayesian rule, *P*(*x^i^* | *α*) can be transformed into *P*(α | *x^i^*), a maximum *a posteriori* (MAP) estimate, where *α* is the estimated parameter. We employ BCS method in our later data acquisition model.

### 2.2. Traditional Hierarchical Routing Method

As pointed out in the previous section, a compressive sensing model can be used in most single-hop WSNs [1,2] or centralized environment [5,22]. In what follows, we present a method for gathering data for a multi-hop WSN in a distributed environment.

To avoid confusion, it is important to note that in this section interpretation of all the variables is involved. Suppose *N* sensors, denoted as *x*_1_, *x*_2_,…,*x_N_*, form a multi-hop route to the sink and the original signal vector is denoted as ***X*** ∈ *R^N^*. Let *d_j_* denote the readings obtained by node *x_j_*. There are two simple data collection schemes: the baseline and compressive methods in a multi-hop route, referred to as Equations (3) and (4), respectively [3]. To avoid excessive overloading in single-tier WSNs, a clustering method has been used in some routing approaches [23,24]. The hierarchical routing method utilizes randomized rotation of local cluster-head nodes to distribute the energy load evenly among the sensors in the network [25]:

(3) $$x_{1}:d_{1}\to x_{2},d_{1}+d_{2}\to x_{3},\ldots ,\sum_{i}^{N-1}d_{i}\to x_{N},\sum_{i}^{N}d_{i}\to \text{sink}$$

(4) $$x_{1}:\sum_{j}^{M}\phi_{j1}d_{1}\to x_{2},\sum_{j}^{M}\phi_{j1}d_{1}+\sum_{j}^{M}\phi_{j2}d_{2}\to x_{3},\ldots ,\sum_{i}^{N}\sum_{j}^{M}\phi_{ji}d_{i}\to \text{sink}$$

### 2.3. Our HRM_CS Model

Inspired by the above theory and method, we put forward our HRM_CS model, which jointly applies the hierarchical routing method and compressive sensing to effectively compress and recover the original signal by exploiting the correlation of sensor readings. Next we describe the HRM_CS model in the formula form. In the HRM_CS model, based on the description of Equation (2), we design a hierarchical routing method to get the value of ***Y*** ∈ *R^M^*. According to LEACH in [4], we divide the *N* nodes into *M* clusters and randomly rotate cluster-heads, where the *M* cluster-head nodes, denoted by {*Y*_1_, *Y*_2_, *Y*_3_,…,*Y_M_*}. However, in order to further reduce energy dissipation and enhance system lifetime, differing from *LEACH*, we only select a part of nodes to transmit data to the cluster head. Assume that {*C*_1_, *C*_2_, *C*_3_,…, *C_M_*} corresponds to the number of nodes in each of the *M* clusters and $\overset{C_{M}}{\underset{c=C_{1}}{\text{∑}}}c=N$. Note that the original signal is ***X*** ∈ *R^N^* and the compressive signal from the *M* clusters is *Y* ∈ *R^M^*.

In order to balance the energy cost among all the nodes, when we create the measurement matrix ***Φ***, in which each row corresponds to the whole *N* nodes in the WSN. We introduce random coefficients *w_k,c_* set to 0 or 1, where *k* denotes the *k*th cluster (*Y_k_*) and *c* denotes the *c*th node (*x_k,c_*) in this cluster. Each sensor contributes its readings to the sink with a non zero coefficient or zero otherwise. And now ***X*** can be expressed as ***X*** = {*x*_1,1_,*x*_1,2_,…,*x*_1,_*_C_*_1_,…,*x_M_*_,1,_*x_M_*_,2,_…,*x_M,CM_*}, where *x_k,c_* is the sensor value and 1 ≤ *k* ≤ *M*, *C*_1_ ≤ *c* ≤ *C_M_*. In this way, a randomly chosen subset of nodes participates in the sensing process so that the lifetime of the whole network is prolonged as long as the energy left in these nodes is higher than the threshold. At first, the sink node picks a random subset of *M* sensors for sampling and broadcasts the selected set of nodes at each round. Then, the selected nodes sample the physical value *x_k,c_* from the sensor. Furthermore, HRM_CS model of jointly applying the hierarchical routing method and compressive sensing process can be formally expressed as Equation (5):

(5) $$\left\{ \begin{array}{l} Y_{K}=\overset{C_{M}}{\underset{c=C_{1}}{\text{∑}}}w_{k,c}x_{k,c}+z_{k,c}\\ Y=\overset{M}{\underset{k=1}{\text{∑}}}Y_{k}+Z_{k}\\ w_{k,c}\sim \text{Gaussian}\;N(0,1)\\ z_{k,c},Z_{k}\sim \text{Gaussian}\;N(0,\beta^{-1}) \end{array}\right.$$

where *z_k,c_* and *Z_k_* denote white Gaussian noise from sensor node *x_k,c_* and cluster *Y_k_*, respectively, and *β*^−1^ denotes the covariance of the noise. Here *Y_k_* and *Y* can act as the superposition of the signal on both the cluster-head node and the sink node. It is clear that the coefficients of ***Φ*** are not fixed at all rounds and the measurement matrix would be changed at each round. Because we compress the data while we transmit the data, we reduce the number of transmissions to the sink, with a corresponding reduction in the energy consumed by the WSN. We provide insight into the data collection based on the hierarchical routing method, which differs from traditional schemes discussed in [3]. Moreover, using the CS technique ensures that the HRM_CS is able to support the online recovery of the original signal ***X*** ∈ *R^N^* from its compressed signal *Y* ∈ *R^M^* with higher accuracy at the sink. It is worth noting that our HRM_CS is not simply about combining the hierarchical routing method and compressive sensing because it is not an efficient and stable way in a real WSNs environment. Next we will analyze the HRM_CS further and give the optimized variant model of HRM_CS.

## 3. Analysis and Optimization of HRM_CS Model

In Section 2 we presented a data acquisition model for WSNs, called the HRM_CS, which combines the hierarchical routing method and compressive sensing. To determine the effectiveness of the HRM_CS and investigate the impact of the routing method on the compression process, we present a formula analysis from multiple aspects. First, without loss of generality, we assume a simple radio model [4], where the radio dissipates *E_elec_* = 50*nJ*/*bit* to run the transmitter or receiver circuitry and *ε_amp_* = 100*pJ*/*bit*/*m*^2^ for the transmission amplifier. The formula to calculate the energy required to transmit a *k*-bit message along a distance of *d*, is given below:

(6) $$E_{Tx}(k,d)=E_{Tx-\text{elec}}(k)+E_{Tx-\text{amp}}(k,d)=E_{\text{elec}}*k+\varepsilon_{\text{amp}}*k*d^{2}$$

In addition, the formula to calculate the energy required to receive a message, is:

(7) $$E_{Rx}(k)=E_{Rx-\text{elec}}(k)=E_{\text{elec}}*k$$

Next we present an analysis of the HRM_CS model from four different aspects.

### 3.1. Sparsity Analysis

There are some common transforms that can be used in a sparse signal model. In this paper, we focus on DCT and PCA. For the DCT, the sparsity depends on the corresponding coefficients with low and middle frequency, whereas for the PCA, the sparsity is determined by the square root of the eigenvalues of the covariance matrix. Note that the default transform in the following section is the DCT.

### 3.2. Measurement Matrix Analysis

We consider two different schemes to build the measurement matrix, *i.e.*, random sampling (RS) and the hierarchical routing method (HRM). The RS scheme is used to determine in a fully distributed way which sensors randomly transmit their data to the sink node at any given time *k*. In the HRM scheme every sensor node first sends its data to local cluster base stations and then these data are transferred to the sink node by the local clusters.

### 3.3. Coherence Analysis between Measurement Matrix and Sparsity Matrix

Coherence analysis between the measurement matrix and sparsity matrix is an important metric that affects the accuracy and stability of information recovery. The formula to compute the coherence μ [15] is given below:

(8) $$\text{Coherence}\;\mu =\underset{i\neq j}{\max}<H_{i},H_{j}>$$

The values of μ depend on a column in the CS matrix, where *H_i_* = ***ΦΨ****_i_*, *H_j_* = ***ΦΨ****_j_*, and $<H_{i},H_{j}>=\Psi_{i}^{T}\Phi^{T}\Phi \Psi_{j}$. From the large number of experiments carried out, we find two methods are able to decrease μ and increase the stability of CS recovery: (1) Adopting the Gaussian random measurement matrix, *i.e.*, the RS scheme. This is because occurrence probability of 0's and 1's in a row of the Gaussian random measurement matrix is relatively fixed so that the coherence being relatively stable; (2) Increasing the number of cluster-head nodes at some extent. This is because that the measurement matrix based on the HRM is built by setting the value of the local head node to “1” and that of the other nodes in this cluster to “0”. Therefore, with an increasing number of cluster-head nodes, both the number of non cluster-head nodes within each new cluster and the total number of occurrences of the value “1” in a row decrease. This leads to the coherence μ decreasing.

### 3.4. Optimization of HRM_CS Model

The HRM_CS objective is to provide a data acquisition method with longer lifetime and higher recovery accuracy under the real WSN environment. In this section, three performance metrics, namely, recovery accuracy (Error), communication cost and compression rate are analyzed. Based on this analysis, the optimized model from the two variant models of HRM_CS, *i.e*., HRM_CS1 with BCS and HRM_CS2 with *L1*-minimization can be obtained. Detailed experimental results are shown in Table 1, where the raw AD values denote the values from the card of Analog signals conversion Digital signals in a real WSN scenario GreenOrbs [18].

First, we investigate recovery accuracy (Error). The max Error is less than 1%, which meets the system demands. Although the HRM_CS1 is a little inferior to the HRM_CS2 with respect to recovery accuracy, HRM_CS1 is superior to the HRM_CS2 with respect to much more stability, *i.e.*, the recovery method based on BCS has greater success than *L1*-minimization (hereafter called *L1*). Moreover, the number *M* of head nodes does not have a large effect on the error and variance of recovery in a BCS scenario. This is because BCS estimates the most probable value by maximizing *a posteriori* without depending on the particular WSN topology considered.

Second, we analyze communication cost. Here we compute the total number of packets in the HRM_CS. The main steps are as follows: (1) *N* − *M* nodes in the cluster send their own sensor readings to *M* cluster-head nodes with an associated cost *O*(*N − M*); (2) Next, superposition of the signal is carried out at the cluster-head node. In addition, the *M* cluster-head nodes transmit their own sensor readings to the sink along a routing path that minimizes the number of transmissions. On this path the packet is not processed but simply forwarded with the longest path *O*(*N*^½^); (3) The total cost of delivering packets to the sink from *M* cluster-head nodes is *O*(*MN*^½^).

Last, we consider compression rate. From a sink point of view, compression rate is defined as $(1-\frac{M}{N})\times 100\%$. It is related to the number *M* of head nodes and increases as *M* decreases, where *N* is the total number of sensor nodes.

From the above analysis, it can easily been seen that the WSN based on the HRM_CS has longer lifetime, compared with the classic approaches given by Equations (3) and (4), where the data are not well compressed. In particular, it can also been seen that the HRM_CS1 with *M* = 40 is our optimal choice since the higher compression rate and stability can be gotten at a little cost of recovery error, compared with the HRM_CS2.

## 4. HRM_CS Framework

In this section we present our framework for implementing the HRM_CS, which performs well for fully distributed compression in WSNs and centralized recovery of an *N*-dimensional signal from a compressed *M*-dimensional signal at the sink. We first present the HRM_CS framework from a holistic viewpoint, and then, we describe WSNs deployment and clustering scenarios. Finally, we introduce the preprocessing for monitoring data, which can optimize the HRM_CS performance.

### 4.1. HRM_CS *Framework for WSNs*

**Figure 1.**
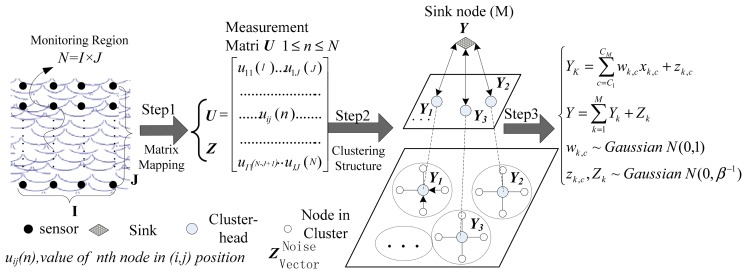
HRM_CS framework.

To implement the model in Equation (5) described in Section 2, we present our framework for WSNs data acquisition in detail. From Figure 1, we can see that the monitoring region is mapped with *N* = *I* × *J* nodes into two-dimensional matrix ***U*** in the first step. Note that ***U*** corresponding to the one-dimensional vector ***X*** in Equation (1). Then, in the next step, we logically re-organize ***U*** to build a clustering structure based on the hierarchical routing method, by randomly selecting cluster-heads and nodes within their clusters to evenly distribute the energy load among the sensors in the network. Each sensor node selected sends its data to local cluster base stations and then these data are transferred to the sink node. In the final step the superposition of the signal is completed and the original signal is recovered at the central sink node based on the optimization techniques.

### 4.2. WSN Deployment

**Figure 2.**
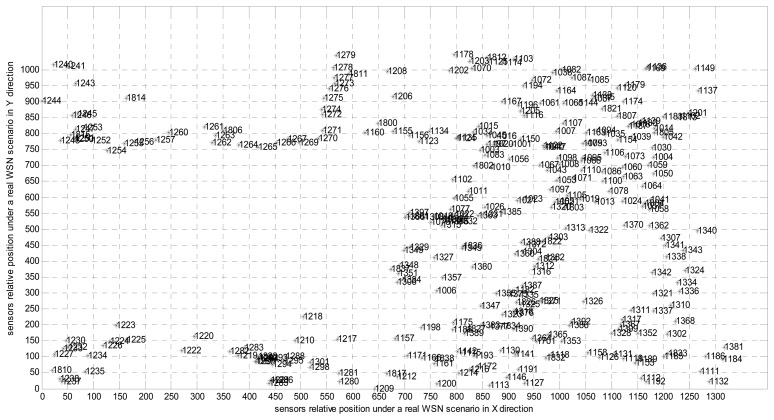
Deployment in a real WSN scenario.

**Figure 3.**
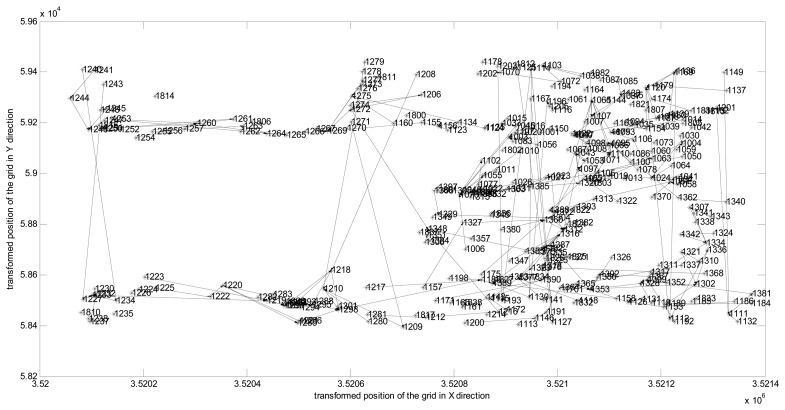
Hierarchical cluster topology in a real WSN scenario.

We consider the deployment in GreenOrbs [18], which is a real WSN scenario set up for long-term monitoring of temperature and humidity in a forest without human supervision. We depict these nodes with their ID and location as shown in Figure 2. The operation of HRM_CS is broken up into rounds, where some node becomes a cluster-head for the current round while each non-cluster-head node decides the cluster to which it will belong for this round. Furthermore, according to the path of the message transmission in one round, we obtain the corresponding screen-shot of hierarchical cluster topology as shown in Figure 3.

### 4.3. Monitored Data Preprocessing

**Figure 4.**
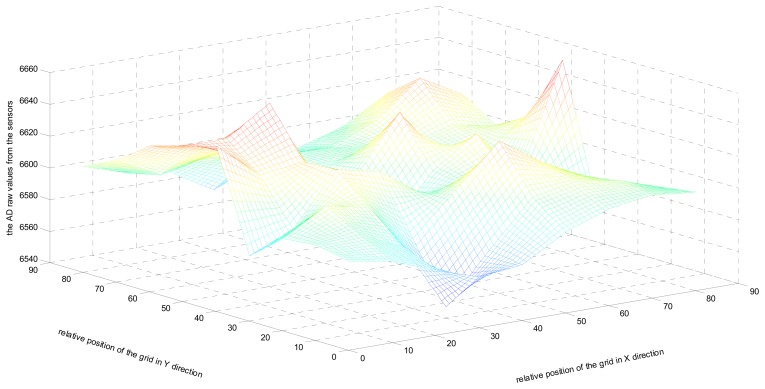
Real monitoring data values displayed in 3D.

In this section, we discuss the preprocessing of the monitored data. As the first step, based on the hierarchical cluster topology as shown in Figure 3, we select the top right region with the most densely distributed nodes as the experimental data. Let the relative position be depicted on the *X*- and *Y*-axis according to the grid structure preprocessing, while the raw AD values from the sensors corresponding to the monitored temperature are given on the *Z*-axis, as displayed in 3D in Figure 4. As is known, the AD raw values from the sensor must be converted into a natural signal temperature by the formula: *temperature* = −39.60 + 0.01 × (*AD raw value*) [18].

**Figure 5.**
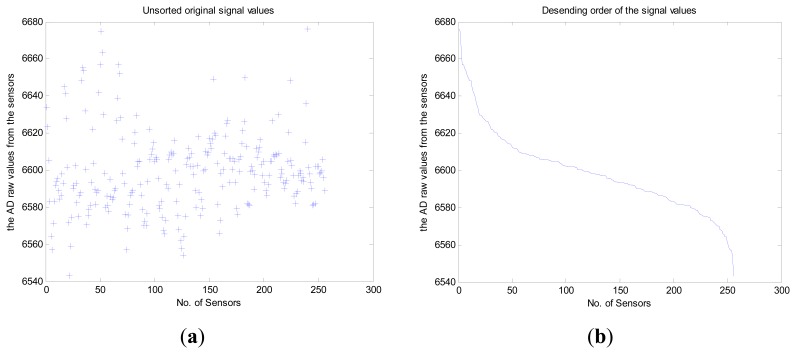
(**a**) Unsorted monitored data values during preprocessing; (**b**) sorted monitored data values during preprocessing.

**Figure 6.**
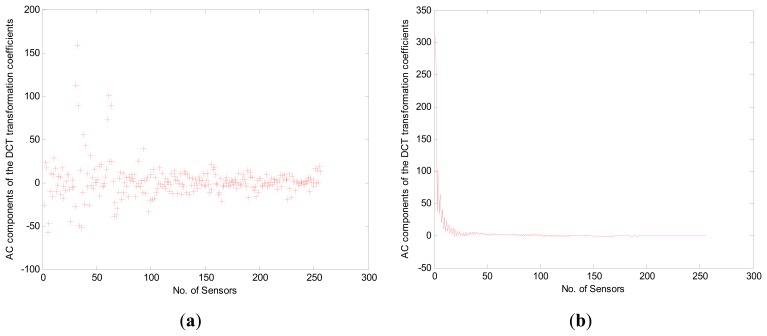
(**a**) Sparsity of unsorted monitored data values during the preprocessing; (**b**) Sparsity of sorted monitored data values during the preprocessing.

Then, in order to optimize the compression rate, we compared two cases: sorted and unsorted monitored data values during the preprocessing. The *X*-axis depicts the 256 nodes while the *Y*-axis gives the raw AD values from the sensors corresponding to the monitored temperature as shown in Figure 5. Note that the compression rate for the “Sorted” case (Figure 5b) is superior to that for the “Unsorted” case (Figure 5a) in the preprocessing since it is affected by a discrete signal characteristic in the frequency domain. To demonstrate the effectiveness of the preprocessing, we create a sparse representation of the “Unsorted” (Figure 6a) and “Sorted” (Figure 6b) monitored data values by DCT. As shown in Figure 6, the *X*-axis depicts the 256 nodes while the *Y*-axis gives the AC components of the DCT transformation coefficients. The “Sorted” case is superior to the “Unsorted” case with respect to compression rate during the preprocessing since “Sorted” case decreases the total communication cost, which coincides with the conclusion in reference [12].

## 5. Performance Comparison

To extensively validate our HRM_CS, we focus on recovery error, energy consumption, and lifetime, compared with other classic WSNs [3,13,25]. As for the limited computational power and communication bandwidth in WSNs, the lifetime of WSNs is the most important metric.

- Energy Consumption
  As the analysis in the previous section shows, compared with the traditional clustering method, the energy consumption of the HRM_CS is substantially reduced by introducing compressive sampling. To analyze the energy consumption, we focus on the sending and receiving phase although there are multiple other phases, such as the sensor computation during the data acquisition. We adopt the energy model given as Equations (6) and (7) in Section 3. For further details refer to [4,26].
- Lifetime
  Assume that each sensor is a tiny powered sensing unit with a finite amount of energy that determines its lifetime. According to the computation energy consumed, when the energy left is lower than zero, we refer to this sensor as dead; otherwise it is alive.
- Recovery Error (*err*)
  Assume an original signal ***X*** ∈ *R^N^*, and recovery signal ***X̃*** ∈ *R^N^*, then:
  (9) $$\text{err}=\frac{{\left\|X-\tilde{X}\right\|}_{2}^{2}}{{\left\|X\right\|}_{2}^{2}}=\frac{\overset{N}{\underset{i=1}{\text{∑}}}{\left(x_{i}-{\tilde{x}}_{i}\right)}^{2}}{\overset{N}{\underset{i=1}{\text{∑}}}x_{i}^{2}}$$

**Figure 7.**
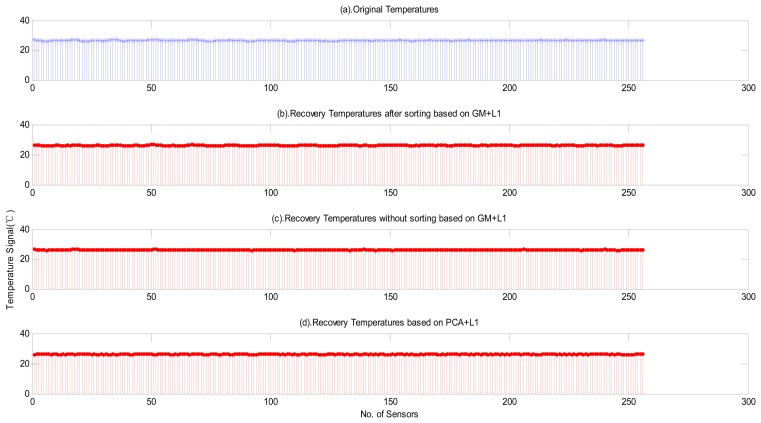
Effects of preprocessing for recovery.

First, let us consider the effects of preprocessing on *L1*-minimization recovery [27]. Figure 7a shows the referenced values after subtracting the minimum value from the original signal. Figure 7b shows the recovery effects with preprocessing and applying a DCT, while Figure 7c shows the recovery effects without preprocessing but applying a DCT. Figure 7d depicts the recovery effects without preprocessing and using PCA. From this experiment, we find that the following results: (1) the recovery error in Figure 7b is smaller than those in Figure 7c,d; (2) the recovery errors in Figure 7c,d are of the same magnitude, however, from the viewpoint of varying trends, the method using PCA in Figure 7d is inferior to that using DCT in Figure 7c since the DCT transform can characterize the original signal using more coefficients with low and middle frequency.

**Figure 8.**
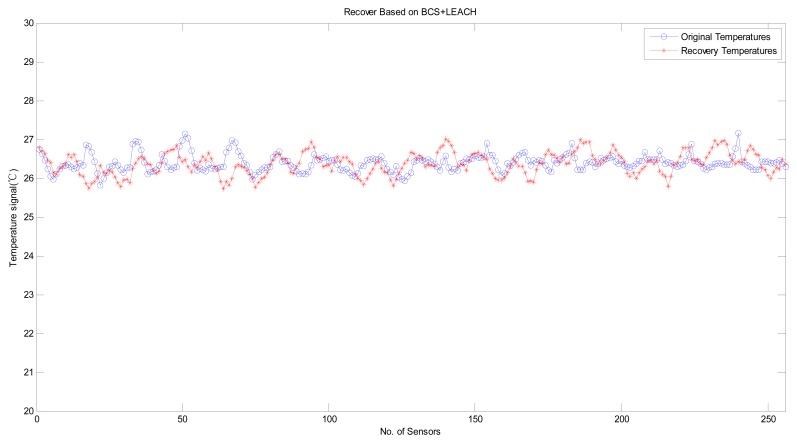
Recovery based on HRM_CS1.

**Figure 9.**
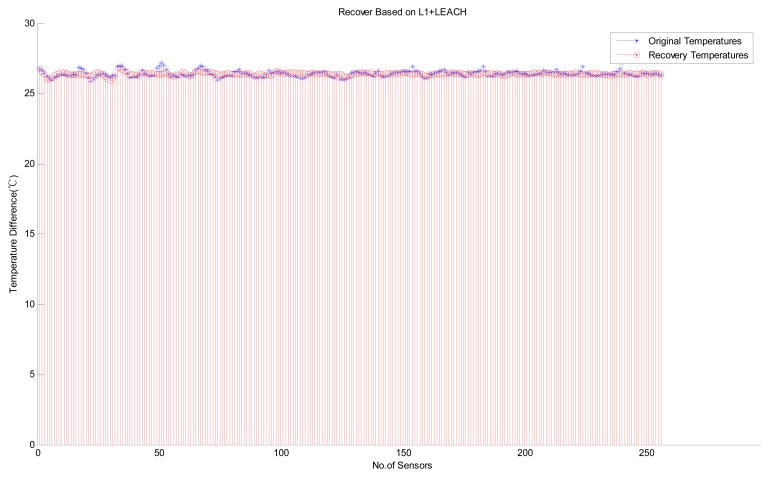
Recovery based on HRM_CS2.

**Figure 10.**
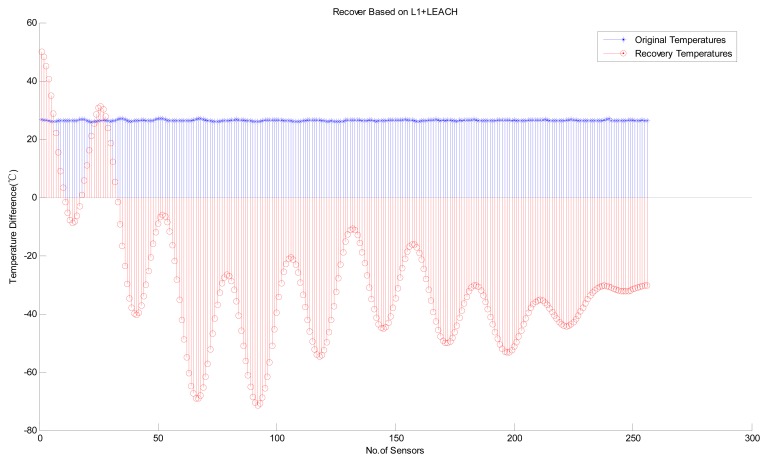
Recovery failure based on HRM_CS2.

Next, we consider the performance of variants of the B HRM_CS model. Figure 8 shows the experimental results of the HRM_CS1, *i.e.*, jointly using CS as the recovery algorithm and LEACH as the hierarchical routing method. Figure 9 depicts the experimental results with high recovery accuracy for the HRM_CS2, *i.e.*, combining *L1*-minimization as the recovery algorithm and LEACH as the hierarchical routing method. Under the HRM_CS2 scenario, if there are too few 1's when creating the measurement matrix, *i.e.*, there are too few nodes to participate in the measurement, it will lead to failure during the recovery operation since the coherence between the measurement and sparsity matrices increases, as shown in Figure 10. Based on the above experiments, as for the stability of information recovery, we find that the applying CS naively like *L1*-minimization may be not proper under the real scenario of WSNs and HRM_CS1 is superior to HRM_CS2.

**Figure 11.**
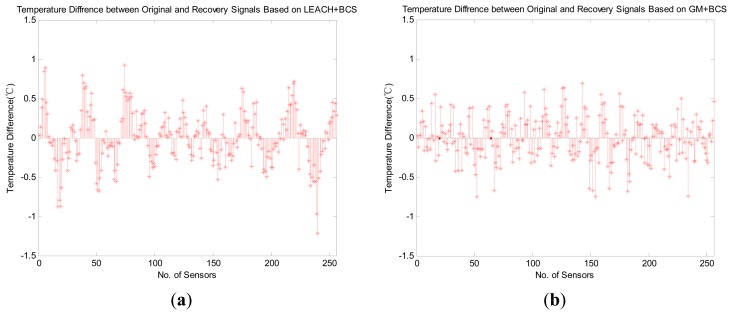
Experimental results of the original and recovered signals with HRM_CS1 and GM_BCS. (**a**) HRM_CS1 mean error = 0.0101; (**b**) GM_BCS mean error = 0.0084.

In Figure 11, we demonstrate the recovery accuracy by comparing the HRM_CS1 with the GM_CS [13] based on the same recovery method, BCS. Figure 11 depicts the experimental results for the original and recovered signals, for which the error is less than 1 °C. Furthermore, the mean error of our HRM_CS1 0.0101 is slightly greater than the mean error of the GM_CS 0.0084 while HRM_CS1 has much longer lifetime than that of the GM_CS, as shown in Figure 13.

**Figure 12.**
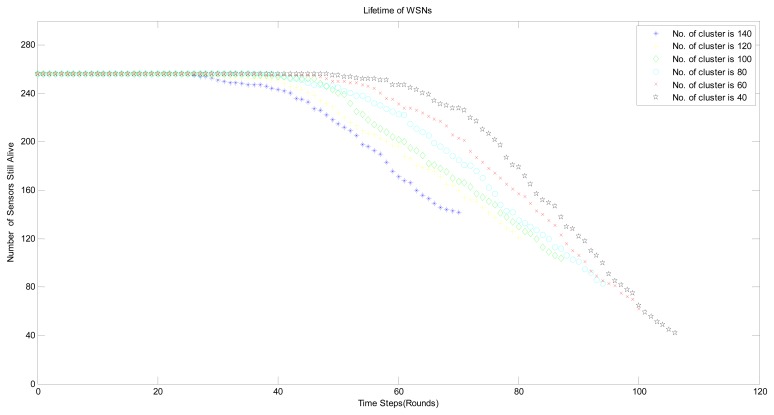
Lifetime of WSNs with different number of clusters.

As for the number of measurements *M*, *i.e.*, the number of clusters in HRM_CS model, it is a vital parameter and has an influence on the lifetime of WSNs. Based on the basic compressive sensing theory [5], we know that *M* ≥ 4*K*, *K* is the number of significant components in original signal ***X*** while *K* varies with the different threshold value during the process of signal, which can be seen in Table 2. As a result, it can be seen from the Figure 12 that the lifetime of WSNs with different number of clusters also take the corresponding changes and the maximal lifetime is obtained at *M* = 40.

**Figure 13.**
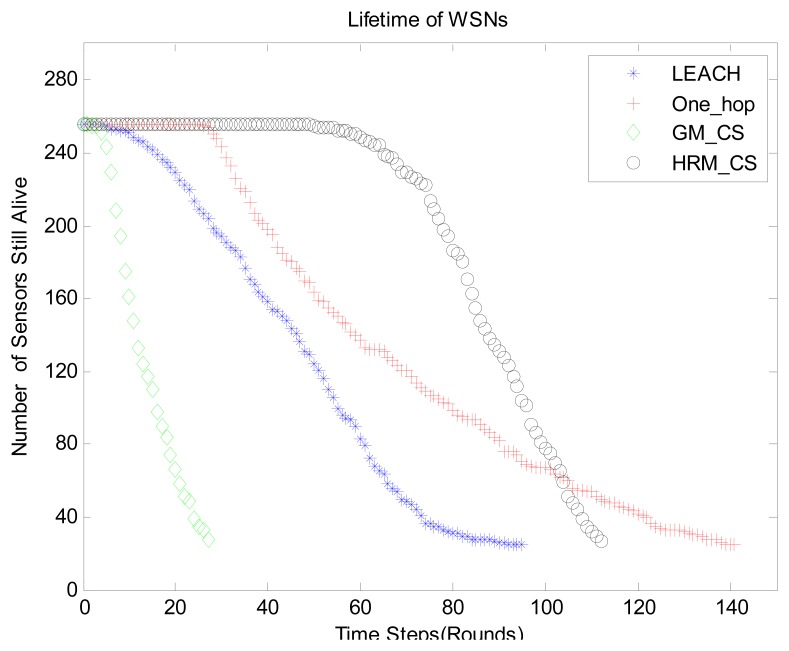
Lifetime of WSNs under different methods.

In the last graph, Figure 13, we show the impact of different data acquisition methods on the lifetime of WSNs. We compared four different methods, namely, HRM_CS, one_hop method [3], traditional LEACH [25] and GM_CS [13]. Here GM_CS means that we use the Gaussian random matrix as the routing matrix jointly with CS. As described in Table 1, *L1*-minimization is more unstable than BCS in this real WSN scenario. Therefore, here we use the BCS as CS recovery algorithm in our experiment. As shown in Figure 13, our solution HRM_CS, is superior to the other methods since the HRM_CS adopts compressive sampling, which decreases the number of communication packets and prolongs the lifetime as a consequence.

## 6. Conclusions

In this paper, we proposed the HRM_CS, a model for data acquisition in WSNs by jointly applying the hierarchical routing method and compressive sensing, which minimizes global energy usage by decreasing the number of samples. HRM_CS outperforms conventional LEACH by introducing the sparse representation technology of PCA and DCT to capture the similarities and differences of the original signal. We studied an approximate recovery of the original signal from this smaller number of WSNs samples with a lower signal reconstruction error, such as *L1*-minimization or Bayesian compressed sensing. Thereafter we compared the performance of these two signal reconstruction techniques combining a different measurement matrix. The extensive validation demonstrates that our solution is effective in reducing energy dissipation and enhancing system lifetime, although it is not necessarily the most accurate. Other supervised WSNs routing algorithms, such as the multicast tree, even for arbitrary topology [12] and routing-free [14], also have attractive features and should be compared when applied jointly with CS in our future work.

## Supplementary Materials

Supplementary materials can be accessed at: http://www.mdpi.com/1424-8220/14/9/16766/s1.

## Figures and Tables

**Table:** 

Symbol	Explanation	Symbol	Explanation	Symbol	Explanation
HRM_CS	Model of combining *HRM* and CS	μ	Coherence between the measurement and sparsity matrices	PCA	Principal Component Analysis
*L1*	Recovery algorithm *L1*-minimization	***Φ***	Measurement matrix	BCS	Bayesian Compressed Sensing
HRM_CS1	Model of combining the *HRM* and the BCS	***Ψ***	Transform Matrix	***X***	One-dimensional data vector
HRM_CS2	Model of combining the *HRM* and the *L1*	*_w__k,c_*	Random Coefficients of *c*th node of *k*th cluster	***Y***	*_M_* random projec-tions vector ***X***
***χ̂***	Estimated value of ***χ***	*x_k,c_*	Sensor value of *c*th node of *k*th cluster	***χ***	K-sparse value of the original signal ***X***

**Table 1.** Performance comparison for HRM_CS.

Recovery Method	Threshold	Number of Cluster Heads *M*	Coherence μ	Success Recovery Rate (%)	Error (%)	Variance
*L1*	50	40	1.950	20	0.262	298.748
BCS	50	40	1.950	100	0.327	466.902
*L1*	40	56	1.495	20	0.247	265.394
BCS	40	56	1.495	100	0.329	472.804
*L1*	35	68	1.085	25	0.224	218.526
BCS	35	68	1.085	100	0.326	463.506
*L1*	28	84	0.658	33	0.250	272.964
BCS	28	84	0.658	100	0.326	463.182
*L1*	25	100	0.556	60	0.209	191.134
BCS	25	100	0.556	100	0.326	462.999
*L1*	20	140	0.388	100	0.222	55.810
BCS	20	140	0.388	100	0.326	462.787

Error: recovery error; Variance: variance of raw AD values between original signal and recovery signal; *L1*: the recovery method based on *L1*-minimization; BCS: the recovery method based on BCS.

**Table 2.** Relationship of *M*, *K* and threshold value.

Threshold Value	Number of Significant Components *K*	Number of Clusters *M*
20	35	140
23	30	120
25	25	100
29	20	80
39	15	60
51	10	40

## Glossary

The table of the key letters and abbreviations in the paper.
